# Supplementary material for: Theileria annulata histone deacetylase 1 (TaHDAC1) initiates schizont to merozoite stage conversion
Source: Sci Rep. 2022 Jul 26;12:12710. doi: 10.1038/s41598-022-15518-7 (PMC9325746; doi:10.1038/s41598-022-15518-7)

Supplementary Table 1: List of *Theileria annulata* genes verified by qRT-PCR and presented in Fig. 2A. FR235222-target genes in *Toxoplasma gondii* were identified by Bougdour et al, (2009).

Gene category	Gene ID	Expressed sequence tag	*Toxoplasma* orthologue ID
***Theileria annulata* orthologues of *Toxoplasma gondii* FR235222 target genes** (n=13)	TA03055	Macroschizont	TGME49_230460
	TA05900	No EST	TGME49_309980
	TA06500	Merozoite, Piroplasm	TGME49_308960
	TA07295	Piroplasm	TGME49_294600
	TA07655	Macroschizont	TGME49_211430
	TA11375	Macroschizont, Merozoite	TGME49_319988
	TA11395	No EST	TGME49_275360
	TA15060	Macroschizont, Merozoite, Piroplasm	TGME49_226310
	TA16180	No EST	TGME49_205750
	TA16310	Merozoite	TGME49_230020
	TA16570	Merozoite, Piroplasm	TGME49_242110
	TA17985	No EST	TGME49_206650
	TA19895	Macroschizont	TGME49_265542
**Random genes** (n=17)	TA02685	Macroschizont, Piroplasm	
	TA02980	Merozoite	
	TA04560	Macroschizont	
	TA06655	Macroschizont, Merozoite	
	TA07550	Merozoite, Piroplasm	
	TA08425	Macroschizont, Merozoite	
	TA09495	No EST	
	TA09590	Macroschizont	
	TA09760	Macroschizont	
	TA09995	No EST	
	TA10915	Macroschizont, Merozoite	
	TA11405	Macroschizont	
	TA13175	Macroschizont	
	TA16685	Merozoite	
	TA18055	Macroschizont, Merozoite, Piroplasm	
	TA19600	Macroschizont, Merozoite	
	TA19860	Macroschizont	

Supplementary Table 2: Effect of apicidin treatment on parasite differentiation potential of infected cloned cell line D7 at 37°C and 41°C.

Anti-Tamr1 +ve cells in drug treated Day 7 37°C cultures		
Treatment	% +ve	Fold increase (Apicidin/DMSO)
Apicidin 25nM	0	0
DMSO	0	
**Anti-Tamr1 +ve cells in apicidin treated Vs DMSO 41°C Day 7 cultures**		
Treatment	% +ve	Fold increase (Apicidin/DMSO)
Apicidin 25nM	28.4*	1.44
DMSO	19.7	

* Significant difference between drug and control culture (P<0.01).
Immunofluorescence was performed as described in[40]; quantification of differentiation and statistical analysis using χ^2 test was as described in[41].

Figure S1. Genomes of medical and veterinary important apicomplexan genera possess a highly conserved histone deacetylase (HDAC1).

```
Cp      -----MAKRVSYFYDGDIGSYYYGPGHPMKPQRIRMAHNLILSYDLYKHMEIYKPHKSPQ        55
Pf      ---MSNRKKVAYFHDPDIGSYYYGAGHPMKPQRIRMTHSLIVSYNLYKYMEVYRPHKSDV        57
Tg      MALSALRKRVAYFYDPDIGSYYYGPGHPMKPQRIRMAHALVLSYDLYKHMEVYRPHKSIE        60
Nc      MALSPLRKRVAYFYDPDIGSYYYGPGHPMKPQRIRMAHALVLSYDLYKHMEVYRPHKSIE        60
Bb      -----MEKRVSYFYDPDVGSYYYGPGHPMKPQRIRMAHALVLSYDLYRHMEVFRPHKAVE        55
Cf      -----MEKRVSYFYDPDVGSYYYGPGHPMKPQRIRMAHALVLSYDLYRHMEIFRPHKAVE        55
Ta      -----MEKRVSYFYDPDVGSFYYGPGHPMKPQRIRMAHALVLSYDLYRHMEIFRPHKAVE        55
Tp      -----MDKRVSYFYDPDVGSFYYGPGHPMKPQRIRMAHALVLSYDLYRHMEIFRPHKAVE        55
               *:*:**:* *:**:*** ***********:* *::**:**::**:::***:

Cp      SELVYFHEEDYINFLSSINPDNSKDFGLQLKRFNLGETTDCPVFDGLFEFQQICAGGSID        115
Pf      NELTLFHDYEYIDFLSSISLENYREFTYQLKRFNVGEATDCPVFDGLFQFQQSCAGASID        117
Tg      PELCLFHSSDYISFLSSVSPENYKEFSLQLKNFNVGEATDCPVFDGLFTFQQACAGASID        120
Nc      PELCLFHSSDYISFLSSVSPENYKEFSLQLKNFNVGEATDCPVFDGLFTFQQACAGASID        120
Bb      PELLAFHDHEYLQFLSGVSPDNYRDFAYQLKRFNVGEATDCPVFDGLYVFQQSCSGASID        115
Cf      PELLSFHDSEYVHFY---------------LRFNVGEATDCPVFDGLYVFQQSCSGASID        100
Ta      PELLSFHDSEYVHFLSGVSPENYRDFTYQLKRFNVGEATDCPVFDGLYVFQQSCSGASID        115
Tp      PELLSFHDSEYVHFLSGVSPENYRDFTYQLKRFNVGEATDCPVFDGLYVFQQSCSGASID        115
         **  **. :*: *                 .**:**:*********: *** *:*.***

Cp      GAYKLNNEQSDICINWSGGLHHAKRSEASGFCYINDIVLGILELLKYHARVMYIDIDVHH        175
Pf      GASKLNHHCADICVNWSGGLHHAKMSEASGFCYINDIVLGILELLKYHARVMYIDIDVHH        177
Tg      AAKKLNHHQADICVNWSGGLHHAKRSEASGFCYINDIVLGILELLKYHARVMYIDIDIHH        180
Nc      AAKKLNHHQADICVNWSGGLHHAKRSEASGFCYINDIVLGILELLKYHARVMYIDIDIHH        180
Bb      GAHRLNNQQADISINWSGGLHHAKRSEASGFCYLNDIVLAILELLKYHARVMYIDIDVHH        175
Cf      AAHRLNNQQADICINWSGGLHHAKRSEASGFCYLNDIVLGILELLKYHARVMYIDIDVHH        160
Ta      AAHRLNNQQADICVNWSGGLHHAKRSEASGFCYLNDIVLGILELLKYHARVMYIDIDVHH        175
Tp      AAHRLNNQQADICVNWSGGLHHAKRSEASGFCYLNDIVLGILELLKYHARVMYIDIDVHH        175
        .* :**:. :**.:********** ********:*****.*****************:**

Cp      GDGVEEAFYLSHRVLTVSFHKFGEFFPGTGDITDIGVAQGKYYSVNVPLNDGIDDDSFLS        235
Pf      GDGVEEAFYVTHRVMTVSFHKFGDYFPGTGDITDVGVNHGKYYSVNVPLNDGMTDDAFVD        237
Tg      GDGVEEAFYVSHRVMTVSFHKFGDFFPGTGDVTDVGASQGKYYAVNVPLNDGMDDDSFVA        240
Nc      GDGVEEAFYVSHRVMTVSFHKFGDFFPGTGDVTDVGASQGKYYAVNVPLNDGMDDDSFVA        240
Bb      GDGVEEAFYVTHRVMTVSFHKFGNFFPGTGDVTDVGVASGKYYSVNVPLNDGMDDESFVD        235
Cf      GDGVEEAFYVTHRVMTVSFHKFGNFFPGTGDVTDVGVSSGKYYSVNVPLNDGIDDESFIE        220
Ta      GDGVEEAFYVTHRVMTISFHKFGNFFPGTGDVTDVGVSSGKYYSVNVPLNDGIDDESFVD        235
Tp      GDGVEEAFYVTHRVMTISFHKFGNFFPGTGDVTDVGVSSGKYYSVNVPLNDGIDDESFVD        235
        *********::***:*:******::******:**:*.  ****:********: *::*:

Cp      LFKPIISKCIEVYRPGAIVLQCGADSVRGDRLGRFNLSIKGHAECVEFCKIFNIPLLILG        295
Pf      LFKVVIDKCVQTYRPGAIIIQCGADSLTGDRLGRFNLTIKGHARCVEHVRSYNIPLLVLG        297
Tg      LFKPVITKCVDVYRPGAIVLQCGADSLTGDRLGKFNLTIKGHAACVAFVKSLDIPLLVLG        300
Nc      LFKPVITKCVDVYRPGAIVLQCGADSLTGDRLGKFNLTIKGHAACVAFVKSLDIPLLVLG        300
Bb      MFRTVVGKCVEVYEPGAIVLQCGADSLTGDRLGRFNLTNKGHAGCVAFCRSLNIPLLVLG        295
Cf      LFKIVIGKCVDVYCPGAIVLQCGADSLTGDRLGRFNLTIKGHAACVDYVKSLNIPLLVLG        280
Ta      LFKVVVGKCVEVYCPGAIVLQCGADSLTGDRLGRFNLTIKGHAACVQYVRSLNIPLLVLG        295
Tp      LFKVVVGKCVEVYCPGAIVLQCGADSLTGDRLGRFNLTIKGHAACVQYVRSLNIPLLVLG        295
        :*: :: **::.* ****::******: *****:***: **** ** . :  :****:**

Cp      GGGYTIRNVARTWAYETATILDRTDLISDNIPLYDYYDYFAPDFKLHIPPLNLPNMNSPE        355
Pf      GGGYTIRNVSRCWAYETGVVLNKHHEMPDQISLNDYYDYYAPDFQLHLQPSNIPNYNSPE        357
Tg      GGGYTIRNVARCWAYETGVVLDRHREMSPHVPLNDYYDYYAPDFQLHLTPSSIPNSNSPE        360
Nc      GGGYTIRNVARCWAYETGVVLDRHREMSPHVPLNDYYDYYAPDFQLHLTPSSIPNSNSPE        360
Bb      GGGYTIRNVARCWAYETGVVLDKHNEMAEQISLNEYYDYYAPDFNLHLQPTNMPNYNTSE        355
Cf      GGGYTIRNVARCWAYETGVILNKHDDMSNQISLNDYYDYYAPDFQLHLTPSSMINYNTPE        340
Ta      GGGYTIRNVARCWAYETGVILNKHTDMSNQISLNDYYDYYAPDFQLHLTPSQMTNYNTKE        355
Tp      GGGYTIRNVARCWAYETGVILNKHTDMSNQISLNDYYDYYAPDFQLHLTPSQMTNYNTKE        355
        *********:* *****..:*::   :  :: * :****:****:**: * .: * *: *

Cp      HLEKIKAKVIDNLRYLEHAPGVEFAYVPSDFFDREASNLQKQEDEEREEELSSWQGGGRA        415
Pf      HLSRIKMKIAENLRHIEHAPGVQFSYVPPDFFNSDIDD----ESDKNQYELKDDSGGGRA        413
Tg      HLEKIKTRVLSNLSYLEHAPGVQFAYVPPDFFGEDNDD----EDEFMQNQVDNEG-GGRA        415
Nc      HLEKIKTRVLSNLGYLEHAPGVQFAYVPPDFFGEDNDD----EDEFMQNQVENEG-GGRA        415
Bb      HLDRIKMKIIENLRHVERAPGVQFAHVPNDFFQYDD-D----EDEAAQLEVFDEG-GGVA        409
Cf      HLDKIKIKILDNLRHVEKAPGVQFAHVPTDFLNIND-D----EDEILQTQIFDES-GGIN        394
Ta      HLDKIKVKILDNLRYVEKSPGVQFAHVPADFLTRDD-D----VDEDLQKQIFDEG-GGIT        409
Tp      HLDKIKVKILDNLRYVEKSPGVQFAHVPPDFLTRDD-D----VDDDLQKQVFDEG-GGRT        409
        **.:** :: .** ::*::***:*::** **:  :  :     .:  : :: .   **

Cp      AGSTESQGNHNEKPKSSRKLQKEHASEFY-----------      444
Pf      PGTRA-----KEHSTTHHLRRKNYDDDFFDLSDRDQSIVPY      449
Tg      AGATAHTA----ANAPYRIRRKDYANDFEDMADRDQKVPI-      451
Nc      AGASTAT-----SAAPYRIRRKDYANDFEDMADRDQKVPI-      450
Bb      PAIVPHRKTTPPIYVNHRLRRKDYKNDYYHLPDRDQHIPI-      449
Cf      NLK-KKYTKTPPIYSFHKLRRKDDKYDFTDLPDRDQNIPI-      433
Ta      TLS-TRK-RVSLMT-THRLRRRDNKGEFYDLPDRDESIPL-      446
Tp      SLS-TRK-RISLITSSHKLRRRDYKGELYDLPDRDDQIPI-      447
                             :  :::    :
```

FR235222 target site

Legend Figure S1. Protein sequence alignment of HDAC1 from several apicomplexan genera. Bb, *Babesia bovis*; Cf, *Cytauxzoon felis*; Cp, *Cryptosporidium parvum*; Nc, *Neospora caninum*; Pf, *Plasmodium falciparum*; Ta, *Theileria annulata*; Tp, *Theileria parva*; Tg, *Toxoplasma gondii*. Multiple sequence alignment was done by Clustal Omega online (https://www.ebi.ac.uk/Tools/msa/clustalo/).

Figure S2. Proliferation of non-infected bovine B cells (BL20) undergoing FR235222 treatment.

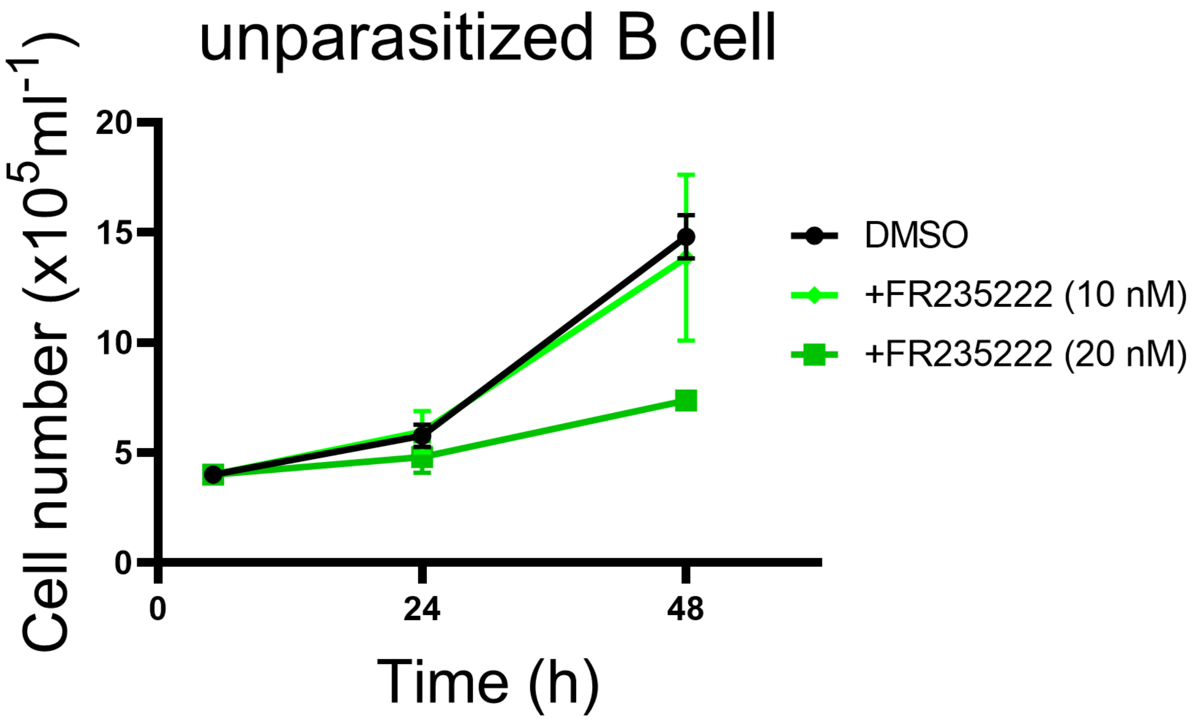

Legend Figure S2. BL20 B cells constantly proliferate, as they are immortalized (but not transformed) by bovine leukemia virus (BLV).

Figure S3. FR235222 treatment and cell viability of *Theileria*-transformed leukocytes

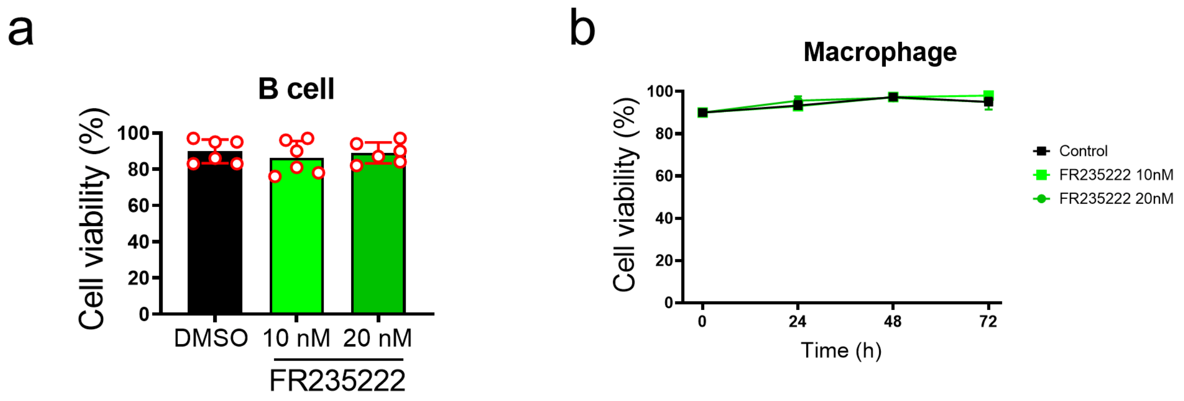

Legend Figure S3. **a** Viability measurement of TBL20 B cells post 48 h exposure to 10 and 20 nM FR235222 compared to DMSO-only control TBL20. **b** Viability of virulent Ode macrophages (p52) throughout 72 h treatment with FR235222 TaHDAC1 inhibitor**.** Each graph is representative of several independent experiments.

Figure S4. Hyperacetylation of *Theileria annulata* histone 4 following TaHDAC1 inhibition by FR235222.

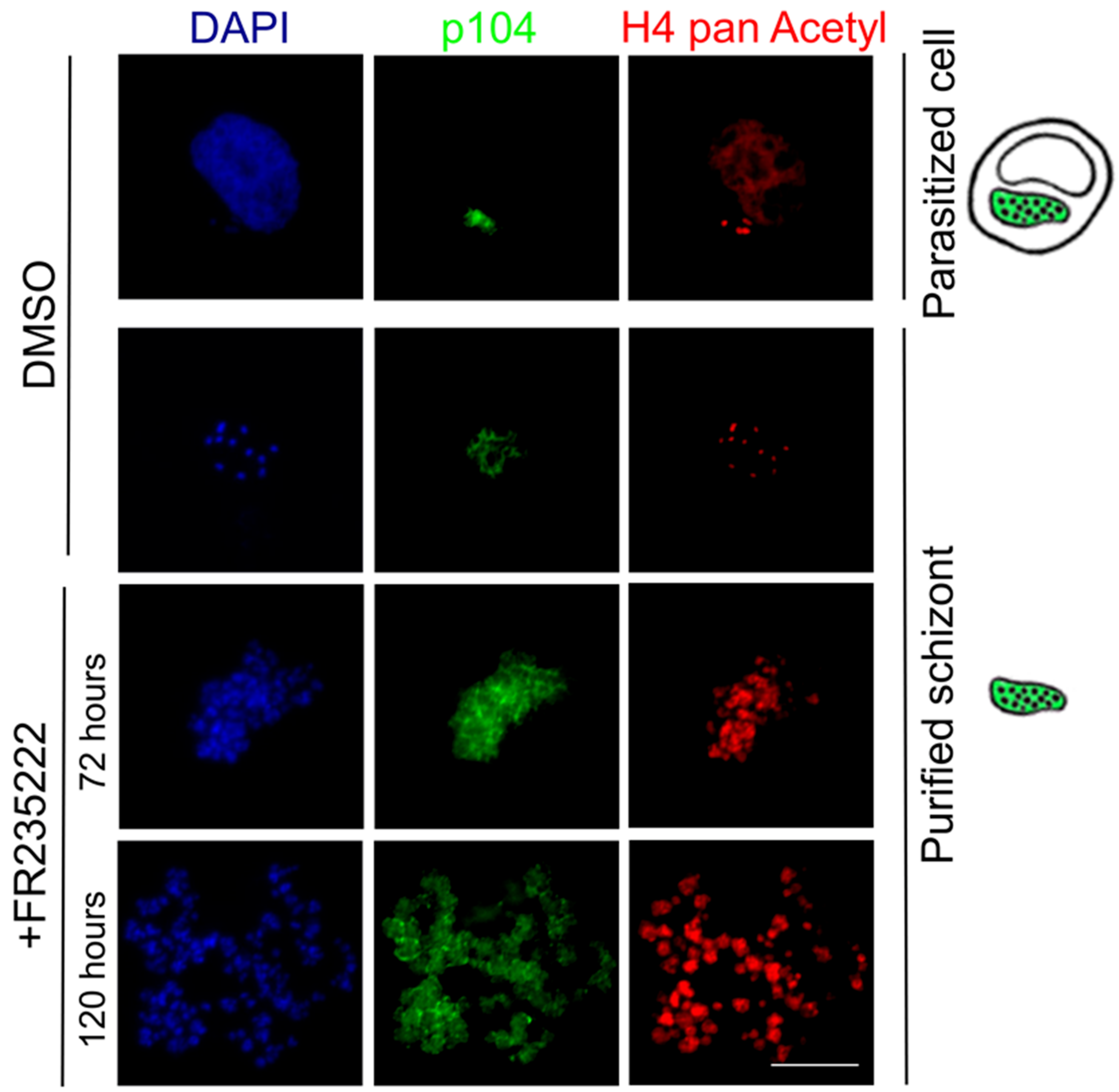

Legend Fig. S4. Immunofluorescence images of TBL20 B cells treated with FR235222. Top row shows a non-treated control. The other rows show parasites purified from treated and control cultures. Parasites were decorated with the 1C12 monoclonal antibody to p104. A rabbit monoclonal antibody detects acetylation of histone 4 at K5, K8 and K12 residues. Host and parasite nuclei stained with DAPI dye. Note the increase in parasite nuclei and H4 acetylation upon FR235222 exposure. X100 magnification, scale bar = 10 µm.

Figure S5. Indirect immunofluorescence using mAb 1D11 that detects merozoite antigen TamR1, or mAb 1C12 that detects the macroschizont p104 antigen.

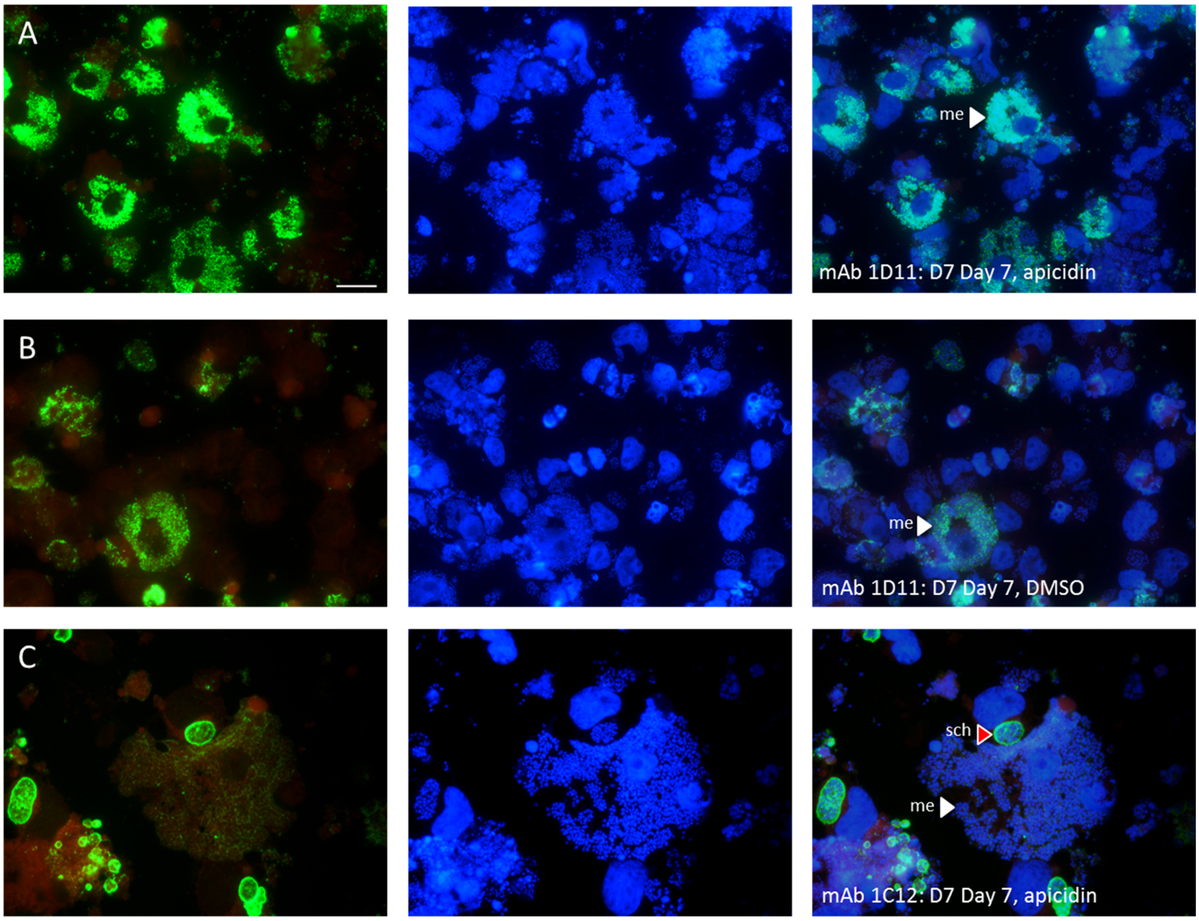

Legend Fig. S5. a and **c** row represent cells derived from culture incubated with apicidin 25 nM; **b** row DMSO control culture. Left column is green image of mAb antibody staining; middle shows DAPI stained nuclei; right column is a merge of first two images. White arrow heads denote cells undergoing merogony, red arrow head denotes macroschizont, scale bar = 10 µm.

Figure S6. Chromosome location of FR235222-induced differentially expressed (DEG) genes.

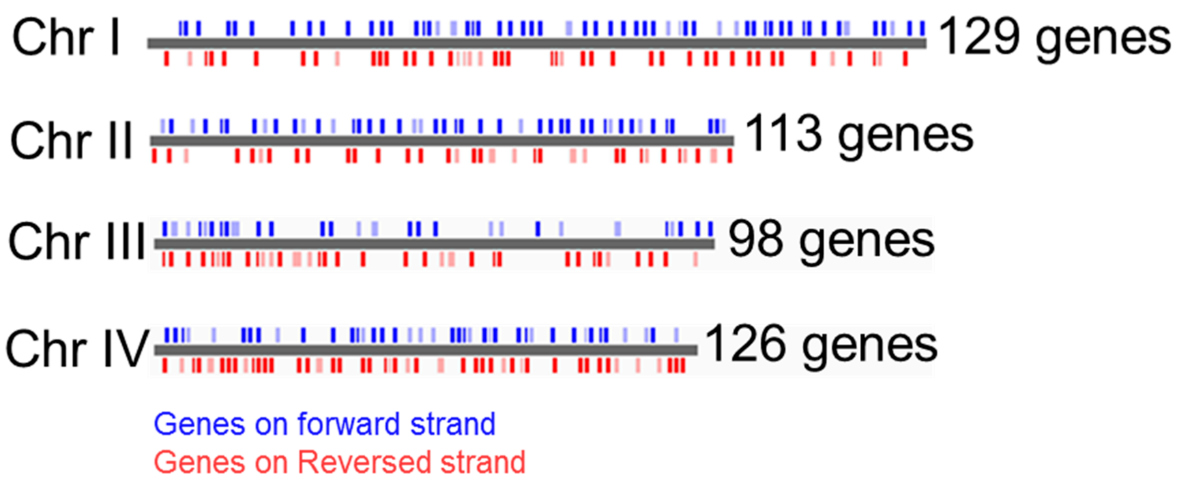

Legend Fig. S6. Chromosome location of 468 (441 up- plus 27 downregulated DEG genes) FR235222-induced differentially expressed genes positioned across the four *T. annulata* chromosomes. Image generated at PiroplasmaDB (https://piroplasmadb.org/piro/app) using the genome viewer feature. Note that two DEGs are mitochondrial and thus not shown.

Figure S7. FR235222-induced expression of *T. annulata* genes.

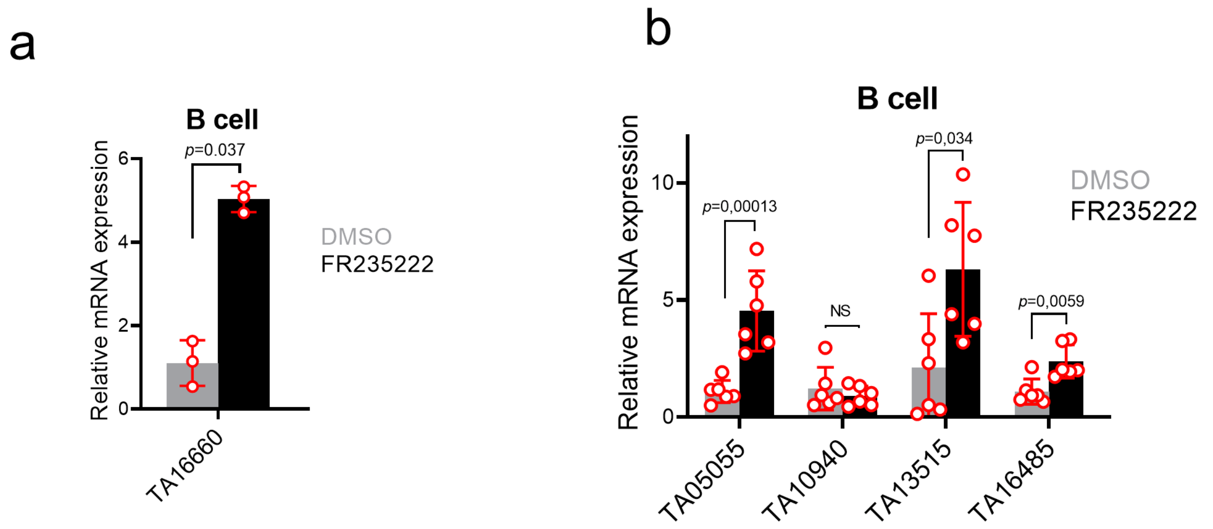

Legend Fig. S7. qRT-PCR results on expression of TA16660 (TaRON) **a** and some AP2 genes **b** in TBL20 cells. Based on RNA-seq data the AP2 genes displayed >3-fold augmentation in expression with non-significant adjusted *p*-values (See Table 3). Results are representative of two biological replicates. *p* values were determined by Student's two-tailed t test. NS: not significant. Error bars depict standard deviations.

Figure S8. Original, unprocessed versions of western blots used to make Figure 2C.

Loading control with anti-Tubulin antibody

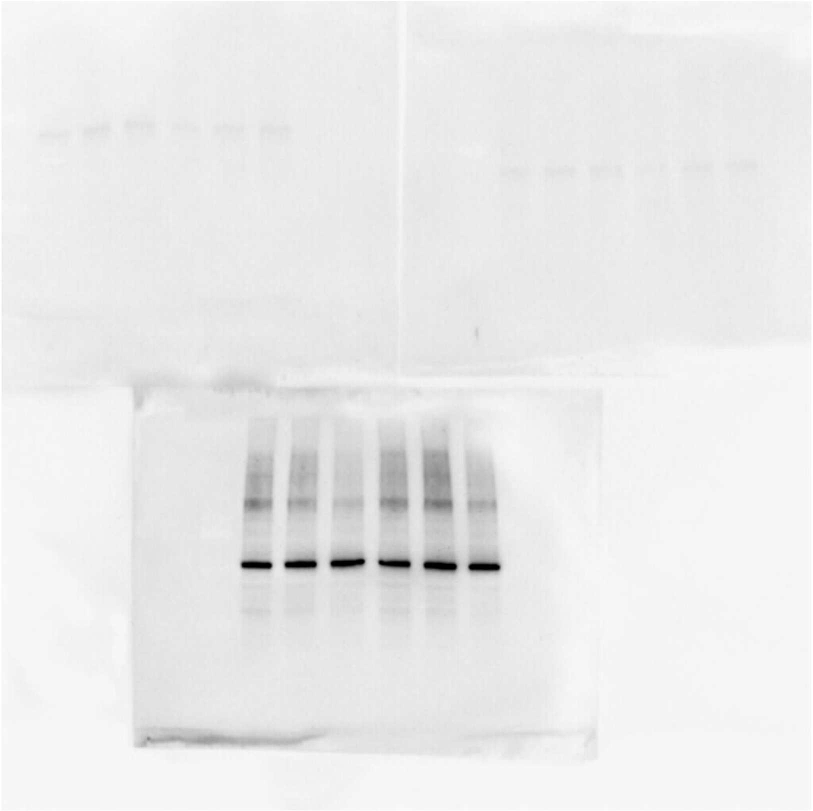

Western blot with anti-TamR1 monoclonal 1D11 antibody

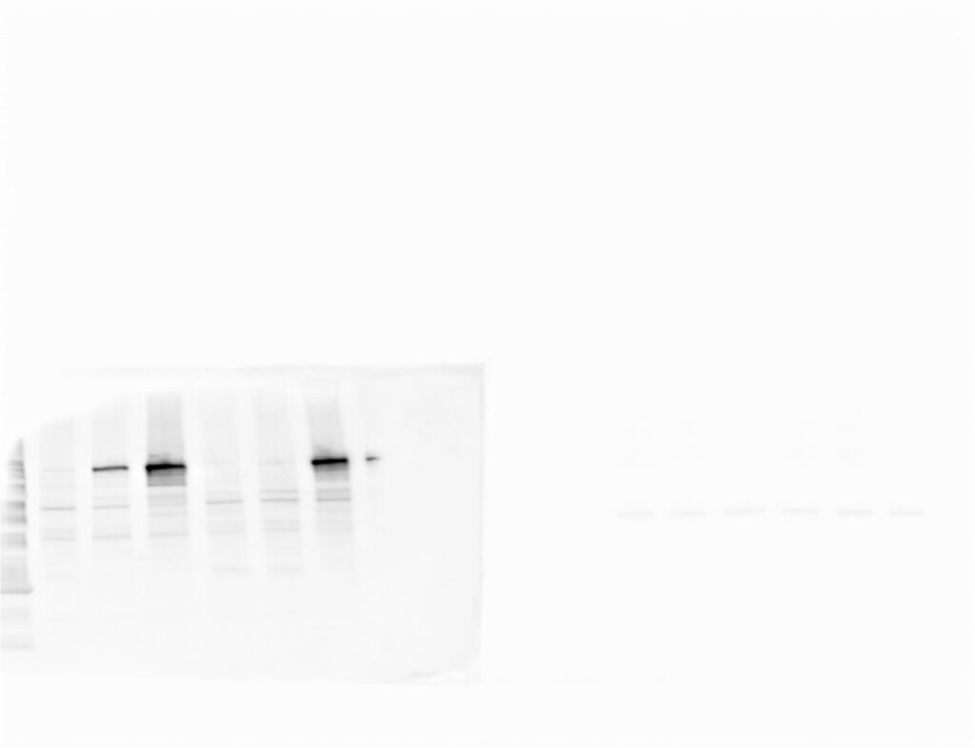

Supplement: Supplementary file 3 — Supplementary Information 3. [file 41598_2022_15518_MOESM3_ESM.pdf]
